# Prognostic nutritional index as an independent risk factor for disease progression in patients with IgA nephropathy

**DOI:** 10.3389/fmed.2025.1530312

**Published:** 2025-02-25

**Authors:** Siqing Wang, Huan Zhou, Lingqiu Dong, Wei Qin

**Affiliations:** ^1^Division of Nephrology, Department of Medicine, West China Hospital of Sichuan University, Chengdu, Sichuan, China; ^2^West China School of Medicine, Sichuan University, Chengdu, Sichuan, China

**Keywords:** IgA nephropathy, prognostic nutritional index, pathologic lesions, renal progression, renal prognosis

## Abstract

**Background:**

Immunoglobulin A nephropathy (IgAN), a common primary glomerulonephritis worldwide, has been investigated, and complex factors are involved in disease progression. A group of evidence emerged that nutrition status plays a nonsubstitutable role in the management of chronic kidney disease. Meanwhile, a novel marker of nutrition and inflammation, the prognostic nutritional index (PNI), has been studied in various diseases. Whether PNI can predict the renal outcome of patients with IgAN remains unclear. Thus, we aimed to evaluate the relationships between PNI and clinicopathologic features, renal progression and renal prognosis in patients with IgAN.

**Methods:**

A total of 1,377 patients with biopsy-proven IgAN were recruited for this retrospective study. All patients were divided into two groups based on the cutoff value of PNI: the high group (PNI ≥ 47.1, *n* = 886) and the low group (PNI < 47.1, *n* = 491). Our study endpoint was end-stage renal disease [estimated glomerular filtration rate (eGFR) < 15 mL/min/1.73 m^2^ or performance of renal replacement therapy]. A correlation test was conducted to explore the relationship between PNI and other important clinicopathologic parameters. The predictive value was determined by the area under the receiver operating characteristic curve (AUROC). Kaplan–Meier and Cox proportional hazards analyses were performed to assess the value of PNI in predicting renal progression and prognosis.

**Results:**

The correlation test revealed that PNI was positively associated with eGFR (*r* = 0.16, *p* < 0.001) and negatively related to 24-h proteinuria (*r* = −0.387, *p* < 0.001). Multivariate Cox regression analysis indicated that low PNI was an independent risk factor for IgAN patients even after adjusting for important clinical and pathological parameters (HR, 0.664; 95% CI, 0.443–0.994; *p* = 0.047). Kaplan–Meier analysis showed that low PNI was significantly correlated with severe renal outcome in patients with IgAN (*p* < 0.001). Moreover, the subgroup analyses of Kaplan–Meier survival demonstrated that low PNI predicted severe renal prognosis in different types of IgAN patients when considering the level of glomerular filtration rate, 24 h proteinuria and hemoglobin.

**Conclusion:**

PNI is associated with renal function and pathologic lesions in IgAN patients and could be a novel marker for the evaluation of renal progression and prognosis.

## Introduction

Immunoglobulin A nephropathy (IgAN) accounts for most primary glomerulonephritis worldwide (1), and approximately 20 to 40% of patients with IgAN reach end-stage renal disease (ESRD) 10 to 20 years after the onset of IgAN, costing considerable socioeconomic resources (2). Chronic inflammation reportedly participates in the pathogenesis of IgAN (3). The therapeutic options for IgAN are limited and remain controversial. Although it is known that various classical factors, such as serum creatine, urine protein, hypertension and pathologic lesions, are predictive of ESRD or short life (4), the clinical course of IgAN may not always be predicted accurately. Therefore, it is always important for clinicians to detect new and effective prognostic markers to better detect high-risk patients for early intervention and reduce the socioeconomic burden of IgAN patients.

The prognostic nutritional index (PNI), which is calculated by multiplying serum albumin and lymphocytes, has been considered as a new prognostic score that reflects both the inflammatory and nutritional conditions of patients and has been found in recent years to be closely associated with prognosis in many diseases. It was reported that higher PNI was linked with longer progression-free survival and overall survival in patients with malignant melanoma and that PNI could be an independent predictor of overall survival in patients with malignant melanoma (5). A previous study found that hypertrophic cardiomyopathy patients with high PNI values had a significantly lower incidence of all-cause mortality and cardiovascular death (6). Moreover, nutritional status was investigated as a predictor of diabetic nephropathy progression (7, 8) and a practical prognostic marker in patients undergoing hemodialysis (9).

However, no studies have been reported on the relationship between PNI and IgAN. Thus, this retrospective study aimed to provide more evidence about the clinical utility of PNI in evaluating the clinicopathologic characteristics of IgAN and predicting the renal prognosis in IgAN patients.

## Materials and methods

### Patients

**FIGURE 1:**
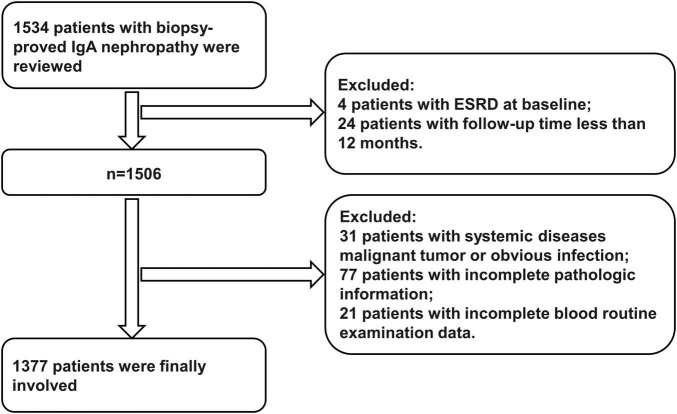
Flowchart of included patients in this study. ESRD, end-stage renal disease.

A total of 1,377 patients with renal biopsy-proven IgAN at West China Hospital of Sichuan University from March 2009 to December 2018 were finally included in our study. Meanwhile, we excluded those patients who missed the standard of this study: (1) Thirty-one patients with systemic diseases, malignant tumors and obvious infection. (2) Seventy-seven patients had incomplete pathologic information. (3) Twenty-one patients had incomplete routine blood examination data. (4) Four patients reached the status of ESRD at baseline. (5) Twenty-four patients had a follow-up time of less than 12 months before reaching the study outcome (Figure 1). Written informed consent for participation in this study was obtained from all patients at baseline. The protocol of this study was performed according to the Helsinki Declaration and authorized by the Ethics Committee of West China Hospital of Sichuan University (2019-33).

### Clinical data

Demographic data (gender, age), medical history (hypertension), examination data (systolic and diastolic blood pressure), and laboratory data [serum creatinine, hemoglobin, lymphocyte count, serum albumin, serum lipid, uric acid, proteinuria, and estimated glomerular filtration rate (eGFR)] were collected before the time of renal biopsy from the hospital electronic information system. Hypertension was defined as blood pressure greater than 140/90 mmHg or the use of antihypertensive agents (10). eGFR was calculated using the CKD-EPI equation (11). The PNI value was evaluated as serum albumin (g/L) + 5 × lymphocyte count (10^9^/L) (12). Anemia was defined as hemoglobin less than 120 g/L in men and less than 110 g/L in women (13).

### Pathological data

Renal biopsy samples were collected and evaluated by light microscopy, especially for hematoxylin–eosin, periodic acid–Schiff, Masson, and periodic acid–Schiff/methenamine staining, immunofluorescence of IgA, IgG, IgM, C3, C4, and C1q, and electron microscopy. The predominance of IgA deposits in the glomerular mesangium, either alone or with IgG, IgM, or complement C3, is the gold standard of IgAN diagnosis (1). According to the Oxford classification of IgAN: mesangial hypercellularity (M0/M1), endocapillary hypercellularity (E0/E1), segmental glomerulosclerosis (S0/S1), tubular atrophy/interstitial fibrosis (T0/T1/T2), and cellular or fibrocellular crescents (C0/C1/C2) (14), experienced pathologists and nephrologists made a unanimous pathological diagnosis.

### Treatment data and endpoints

According to the therapeutic regimen of all patients after renal biopsy, they were divided into a supportive treatment group and a prednisone or other immunosuppressive agent group. For adult non-dialysis CKD patients with Hb concentration < 100 g/l, according to KDIGO guideline, we made the decision to initiate erythropoietin (ESA) therapy be individualized based on the rate of hemoglobin concentration decline, previous response to iron therapy, the risk of needing a blood transfusion, the risks associated with ESA therapy, and the presence of symptoms attributable to anemia, especially the patients with transferrin saturation > 20% and ferritin > 200 ng/ml (15). The study endpoint was end-stage renal disease (ESRD: eGFR less than 15 mL/min/1.73 m^2^ or undergoing renal replacement treatment) and/or an eGFR decrease of more than 50% compared with baseline.

### Statistical analysis

All statistical analyses were performed using IBM SPSS software, version 26.0 (IBM Corp., Armonk, NY, USA) and R version 4.2.1.^1^ For continuous variables, the Kolmogorov–Smirnov test was used to determine whether the study data complied with the normal distribution. For normally distributed data distributed data, means ± standard deviations were used to for expression. Data that did not fit the normal distribution were expressed as medians with interquartile ranges (25th and 75th percentiles), whereas counts and percentages were used for categorical variables. Comparisons of continuous variables between two groups were performed by the independent *t*-test or nonparametric test where appropriate. Chi-squared or Fisher’s exact tests were used to evaluate the differences in the categorical variables. The relationships between the PNI and other important parameters were explored by a correlation test. The discriminatory power of PNI for composite renal outcome was tested by the area under the receiver operating characteristic curve (AUROC), and the prediction of PNI at different times for renal risk was evaluated with a time-dependent receiver operating characteristic (td-ROC) curve. In addition, the optimal cutoff point of the PNI was obtained by conducting a log-rank test. Comparison of renal survival among different groups was evaluated by the Kaplan–Meier method. Cox regression analysis was performed to discriminate the risk factors for renal outcome. Hazard ratios (HRs) estimated from the Cox analysis were reported as relative risks with corresponding 95% confidence intervals (CIs). Statistical significance was considered if *p* < 0.05.

## Results

### Demographic and clinicopathological characteristics

**FIGURE 2:**
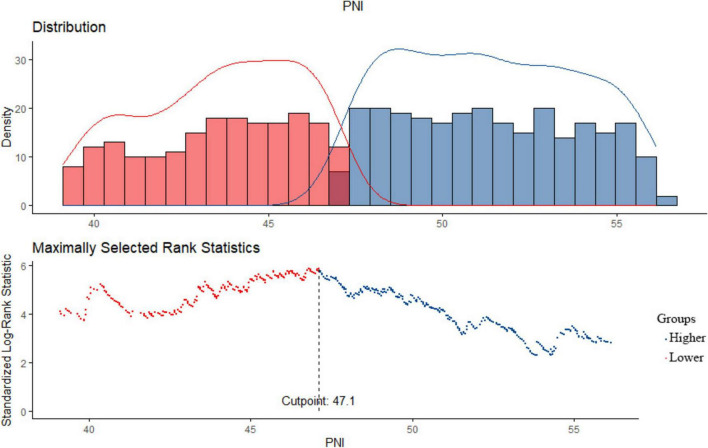
The optimal cutoff value of PNI by the log-rank test.

The baseline data of the 1,377 patients are shown in Table 1. The mean age of all patients was 33 years old, and 45% were male. A total of 27.8% of the patients had a medical history of hypertension. In addition, the median eGFR and proteinuria were 92.47 (65.95–116.53) mL/min/1.73 m^2^ and 1.41 (0.74–3) g/day, respectively. The median PNI was 49.3 (44.7–53.1) in all patients at the time of renal biopsy. Then, we divided all the patients into two groups according to the PNI value by conducting a log-rank test, which revealed that the optimal cutoff PNI to predict the composite renal outcome in patients with IgAN was 47.1 (Figure 2). Thus, the patients were distributed into a high PNI group (PNI ≥ 47.1) and a low PNI group (PNI < 47.1). Compared with the low PNI group, patients with high PNI had a lower incidence of anemia (24.4 vs. 7.9%) and a lower use rate of prednisone or other immunosuppressive agents (76.2 vs. 51%). Additionally, patients with a higher PNI were more likely to have lower levels of proteinuria, serum creatinine, total cholesterol, low-density lipoprotein cholesterol (LDL-C), and high-density lipoprotein cholesterol (HDL-C). With respect to pathologic lesions, we found that patients with higher levels of PNI had a lower rate of renal tubular atrophy/interstitial fibrosis. In our study, there were 43.2, 25.7, 24, and 7.1% patients with CKD stages 1 to 4 in the low group, respectively, while the values were 57.8, 26.5, 13.8, and 1.9% in the high group. Meanwhile, the correlation analyses showed that the PNI was positively correlated with the eGFR (*r* = 0.16, *p* < 0.001) and negatively related to proteinuria (*r* = −0.387, *p* < 0.001).

**TABLE 1:** Demographic and clinicopathological characteristics of 1,377 IgAN patients.

Parameters	All (*n* = 1,377)	Group 1 (*n* = 491) PNI < 47.1	Group 2 (*n* = 886) PNI ≥ 47.1	*P*-value
Male (%)	620 (45%)	169 (34.4%)	451 (50.9%)	< 0.001
Age (years)	33 (26, 42)	35 (26, 44)	32 (26, 41)	0.002
MAP (mmHg)	96.33 (88.67, 105.83)	96.67 (88.67, 106.67)	96 (88.67, 105.33)	0.265
Hypertension (%)	383 (27.8%)	138 (28.1%)	245 (27.7%)	0.857
Anemia (%)	190 (13.8%)	120 (24.4%)	70 (7.9%)	< 0.001
LY (× 10^9^/L)	1.76 (1.42, 2.2)	1.53 (1.21, 1.89)	1.92 (1.59, 2.34)	< 0.001
PNI	49.3 (44.7, 53.1)	42.85 (37.95, 45.05)	51.75 (49.55, 54.6)	< 0.001
Proteinuria (g/d)	1.41 (0.74, 3)	2.35 (1.06, 4.21)	1.05 (0.64, 2)	< 0.001
**Proteinuria classification**				
24 h-proteinuria ≥ 1 g/d	932 (67.7%)	407 (82.9%)	525 (59.3%)	< 0.001
24 h-proteinuria ≥ 3.5 g/d	228 (16.6%)	152 (31%)	76 (8.6%)	< 0.001
URBC (/HP)	19 (7, 62)	24 (9, 80)	16 (5, 53)	< 0.001
ALB (g/L)	40.1 (36.3, 43.4)	34.7 (29.4, 37.3)	42.4 (40.1, 44.7)	< 0.001
SCr (umol/L)	83.7 (65.9, 109.3)	87 (65.9, 126.4)	82 (65.8, 104)	0.004
eGFR (mL/min/1.73 m^2^)	92.47 (65.95, 116.53)	79.49 (52.26, 113.34)	96.15 (74.53, 117.71)	< 0.001
**CKD stages**				< 0.001
Stage 1	724 (52.6%)	212 (43.2%)	512 (57.8%)	
Stage 2	361 (26.2%)	126 (25.7%)	235 (26.5%)	
Stage 3	240 (17.4%)	118 (24%)	122 (13.8%)	
Stage 4	52 (3.8%)	35 (7.1%)	17 (1.9%)	
UA (umol/L)	364 (302, 441)	358 (299, 442)	365.35 (303.68, 441)	0.629
Hyperuricemia (%)	546 (39.7%)	204 (41.5%)	342 (38.6%)	0.284
Hypertriglyceridemia (%)	579 (42%)	222 (45.2%)	357 (40.3%)	0.076
Hypercholesterolemia (%)	512 (37.2%)	249 (50.7%)	263 (29.7%)	< 0.001
LDL-C (mmol/L)	2.72 (2.18, 3.39)	3.03 (2.34, 3.96)	2.63 (2.11, 3.16)	< 0.001
HDL-C (mmol/L)	1.38 (1.11, 1.73)	1.46 (1.17, 1.9)	1.35 (1.09, 1.64)	< 0.001
**Pathological lesions**				
M				0.02
0	339 (24.6%)	103 (21%)	236 (26.6%)	
1	1,038 (75.4%)	388 (79%)	650 (73.4%)	
E				< 0.001
0	1,315 (95.5%)	449 (91.4%)	866 (97.7%)	
1	62 (4.5%)	42 (8.6%)	20 (2.3%)	
S				0.446
0	543 (39.4%)	187 (38.1%)	358 (40.2%)	
1	834 (60.6%)	304 (61.9%)	530 (59.8%)	
T				< 0.001
0	1,072 (79.3%)	358 (72.5%)	736 (83.1%)	
1	235 (17.1%)	105 (21.4%)	130 (14.7%)	
2	50 (3.6%)	30 (6.1%)	20 (2.3%)	
C				0.001
0	1,070 (77.7%)	364 (74.1%)	706 (79.6%)	
1	258 (18.7%)	98 (20%)	160 (18.1%)	
2	49 (3.6%)	29 (5.9%)	20 (2.3%)	
Treatment				< 0.001
Support treatment (%)	551 (40%)	117 (23.8%)	434 (49%)	
Prednisone or other immunosuppressive agents (%)	826 (60%)	374 (76.2%)	452 (51%)	

MAP, mean arterial pressure; LY, lymphocytes counts; PNI, neutrophil-to-lymphocyte ratio; URBC, urinary red blood cell counts; ALB, albumin; SCr, serum creatinine; eGFR, estimated glomerular filtration rate; CKD, chronic kidney disease; UA, uric acid; LDL-C, low-density lipoprotein cholesterol; HDL-C, high-density lipoprotein cholesterol; M, mesangial proliferation; E, endocapillary proliferation; S, segmental sclerosis; T, tubular atrophy/interstitial fibrosis; C, crescents; CKD, chronic kidney disease.

### Predictive value of PNI for renal outcome in IgAN

**FIGURE 3:**
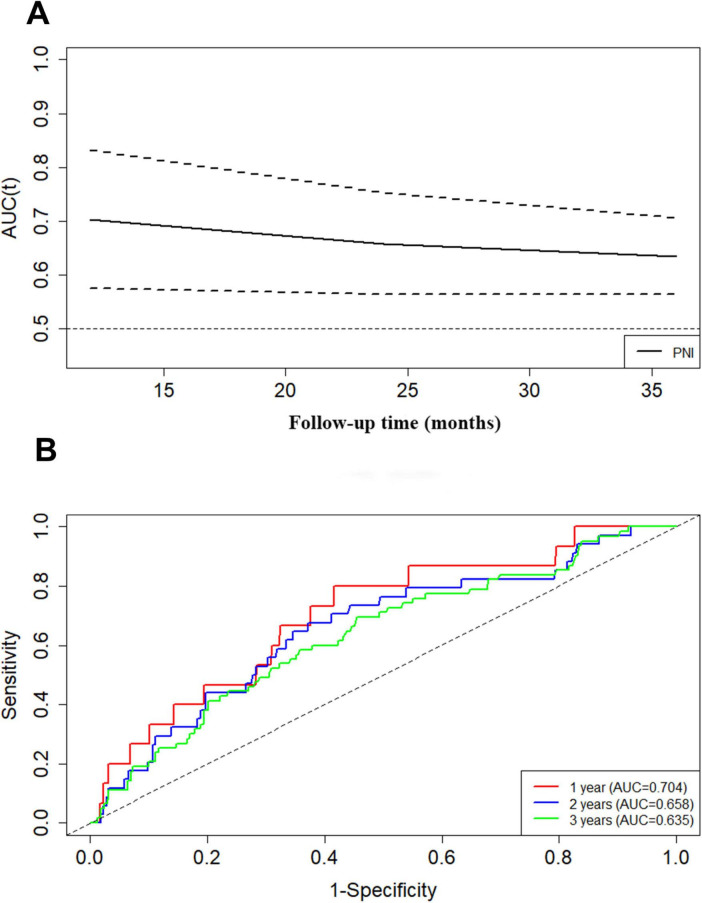
The prediction of PNI at different times for composite renal outcome evaluated with time-dependent receiver operating characteristic curve **(A,B)**.

For the prediction of composite renal outcome, the area under the receiver operating characteristic curve (AUROC) was 0.648 for PNI. In addition, the area under the curve (td-AUC) evaluated with time-dependent receiver operating characteristics was 0.704 at 1 year, 0.658 at 2 years, and 0.635 at 3 years (Figures 3A, B).

### PNI and renal prognosis in patients with IgAN

Univariate Cox regression analysis revealed that a higher PNI revealed a lower composite renal risk (HR, 0.376; 95% CI, 0.268–0.529; *p* < 0.001). Moreover, multivariate Cox regression analysis suggested that high PNI was consistently related to decreased renal risk (HR, 0.664; 95% CI, 0.443–0.994; *p* = 0.047) after adjustment for age, gender, hypertension, e-GFR, urine protein more than 1 g per day, anemia, serum albumin concentration less than 30 grams per liter and the Oxford MEST-C score (Table 2).

**TABLE 2:** Associations of PNI with IgAN progression.

	HR (95% CI)	*P*-value
Univariate	0.376 (0.268–0.529)	< 0.001
Model 1	0.642 (0.430–0.960)	0.031
Model 2	0.658 (0.441–0.983)	0.041
Model 3	0.664 (0.443–0.994)	0.047

PNI was analyzed as a categorial variable (higher PNI vs. lower PNI) and data are reported as hazard ratio (HR) and 95% confidence interval (CI). Model 1: adjusted for baseline age, gender, hypertension, e-GFR, 24 h-proteinuria ≥ 1 g/d, hypoalbuminemia and anemia. Model 2: adjusted for covariates in model 1 plus pathological lesions (MEST-C). Model 3: adjusted for covariates in model 2 plus treatment.

**FIGURE 4:**
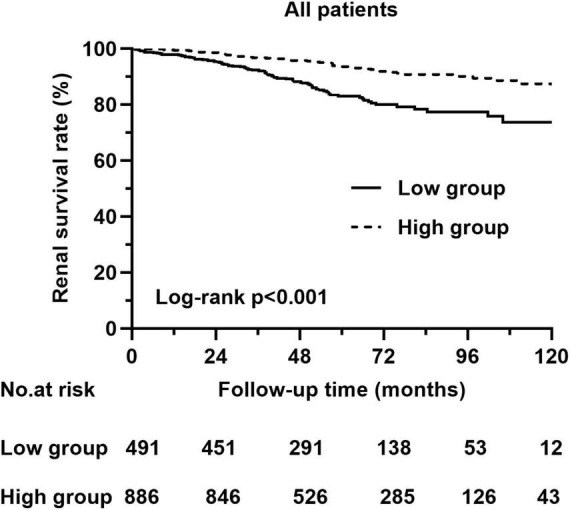
Kaplan–Meier curves of renal survival rate in patients with different PNI levels.

**FIGURE 5:**
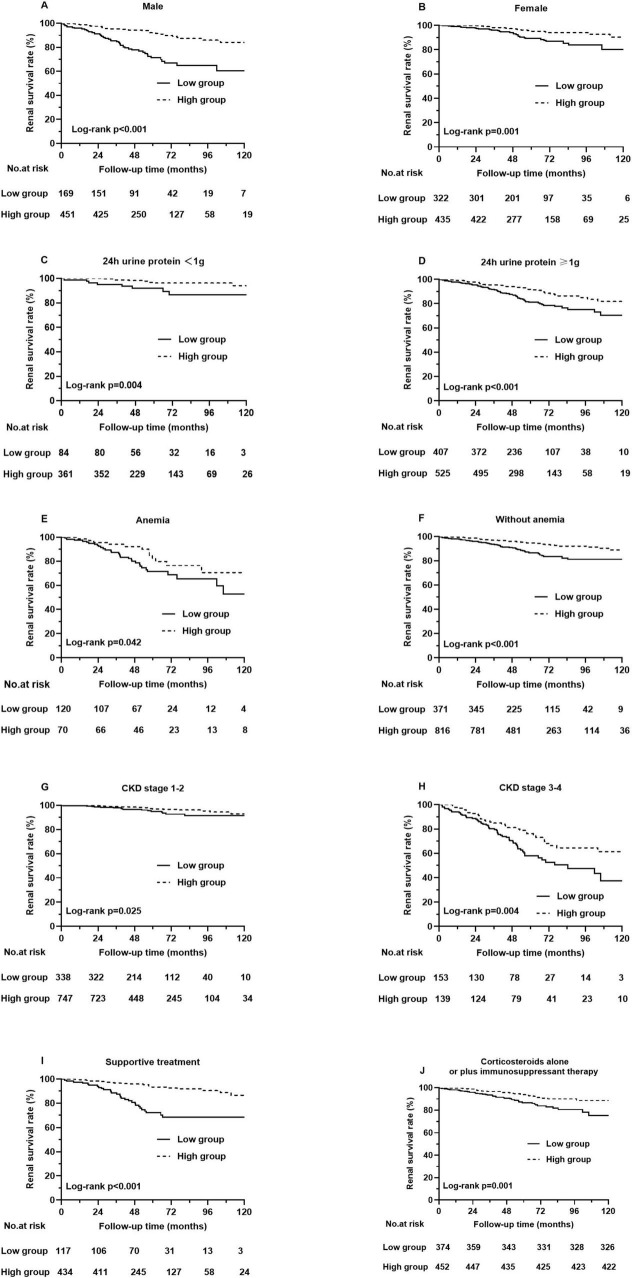
Different types of Kaplan-Meier analysis for the composite renal outcome. **(A)** Renal survival rates in male patients, **(B)** Renal survival rates in female patients, **(C)** Renal survival rates in patients with 24 h urine protein < 1 g, **(D)** Renal survival rates in patients with 24 h urine protein ≥ 1 g, **(E)** Renal survival rates in patients with amenia, **(F)** Renal survival rates in patients without amenia, **(G)** Renal survival rates in patients with CKD stage 1–2, **(H)** Renal survival rates in patients with CKD stage 3–4. **(I)** Renal survival rates in patients with supportive treatment, **(J)** Renal survival rates in patients with corticosteroids alone or plus immunosuppressant therapy.

Kaplan–Meier analysis indicated that patients with higher PNI at baseline had a significantly lower composite renal risk (*p* < 0.001, Figure 4). We next investigated the association of PNI with renal risk in subgroups of patients with different clinical characteristics. The results demonstrated that a higher PNI was still significantly associated with a reduced renal risk in males and females (Figures 5A, B), in patients with or without amenia (Figures 5C, D), in patients with CKD stage 1–2 or CKD stage 3–4 (Figures 5E, F), in patients with proteinuria greater than 1 g/day or not (Figures 5G, H), and in patients with prednisone and/or other immunosuppressive agents or supportive treatment (Figures 5I, J).

## Discussion

In the present study, PNI was shown to be a prognostic indicator with an AUROC of 0.648 in individuals with biopsy-confirmed IgAN. In addition, we observed that patients with a low PNI (PNI < 47.1) were associated with more severe clinical features and pathologic lesions. We also found that PNI was positively correlated with 24-h urine protein and negatively related to eGFR. Besides, our findings suggested that a low PNI is an independent novel prognostic factor for composite renal risk in patients with IgAN after adjusting for important clinicopathological parameters.

The area under the curve (td-AUC) of PNI in IgAN patients evaluated with time-dependent receiver operating characteristics was 0.704 at 1 year, 0.658 at 2 years, and 0.635 at 3 years. This demonstrated that PNI could be a risk predictor for short-term renal outcome for patients with IgAN. Furthermore, in univariate Cox analysis PNI emerged as predictors, and high PNI had an HR of 0.376 compared with low PNI. The results of different multivariate Cox regressions likewise revealed that low PNI constituted a risk factor for renal survival, as patients with this characteristic showed greater renal risk than other patients. In line with these findings, we presumed that the PNI, as a combination of two clinically common and easily detected indicators, is useful in survival prediction.

The renal survival analysis revealed that patients with low PNI had severe renal outcomes. To better understand the value of PNI in predicting renal prognosis, we conducted subgroup analyses based on gender, CKD stage, anemia status, 24-h urine protein level and treatment. Grouped by gender, individuals with a higher PNI tended to have higher renal survival rates than those with a lower PNI in both males and females. In the subgroups of patients with different CKD stages, patients with higher PNI had a lower risk of progression to composite renal outcome in both CKD stage 1–2 and CKD stage 3–4. Furthermore, in the subgroups of patients without anemia, the PNI still predicted the renal risk in IgAN. Similarly, grouped based on clinical features, PNI was still related to renal outcomes in patients with or without 24-h urine protein greater than 1 g and with supportive treatment or with prednisone and/or other immunosuppressive agents. These results indicated that the PNI has wide applicability in predicting renal outcomes in different IgAN patients.

Increasing evidence has demonstrated that hypoproteinemia and persistent proteinuria are the key factors affecting the progression and prognosis of IgAN (16, 17). In our study, we found that low PNI was correlated with more proteinuria. PNI is a measure of combined serum albumin and lymphocyte counts, and patients with low PNI may have low serum albumin, while patients with chronic kidney disease may have low serum albumin due to chronic systemic inflammation or loss of albumin into the urine (18). In recent years, dietary management of IgAN has received more and more attention from researchers and clinicians. Due to the high dietary protein intake can cause intraglomerular hypertension, which may result in kidney hyperfiltration, glomerular injury, and proteinuria. Excessive protein consumption increases the burden on the kidney (19). However, protein is also needed to provide energy for the normal life, so a high-quality low-protein diet has been recommended since a clinical cohort study reported that underweight is an independent risk factor for kidney disease progression in IgAN, which might be associated with malnutrition status (20). Energy intake is crucial, and higher intake is associated with maintaining a neutral or positive nitrogen balance. Adequate nutritional and dietary support are fundamental in preventing nutritional inadequacies and muscle wasting (21). Meanwhile, it was reported that prescribing a very low-protein diet supplemented with ketoanalogues compared with standard low protein diet is safe, but does not provide additional advantage to the kidney or patient survival (22). Besides, it was found that a low protein diet for patients with kidney diseases was significantly associated with depressive symptoms and poor health-related quality of life (23).

Immunoinflammatory state has also been proved to be a key factor affecting the prognosis of kidney disease. In addition, inflammation leads to lymphocyte apoptosis and the downregulation of lymphocyte differentiation and proliferation (24). Furthermore, lymphocytes are related to physiological stress and malnutrition (25). All of these factors in turn could exacerbate inflammation and create a vicious cycle to accelerate disease progression. Thus, PNI could be accepted as a marker of chronic low-grade inflammation. In addition, inflammation is one of the most important initiators of progressive tubulointerstitial fibrosis (26). We also found that patients with higher PNI levels had a lower rate of tubular atrophy/interstitial fibrosis.

**FIGURE 6:**
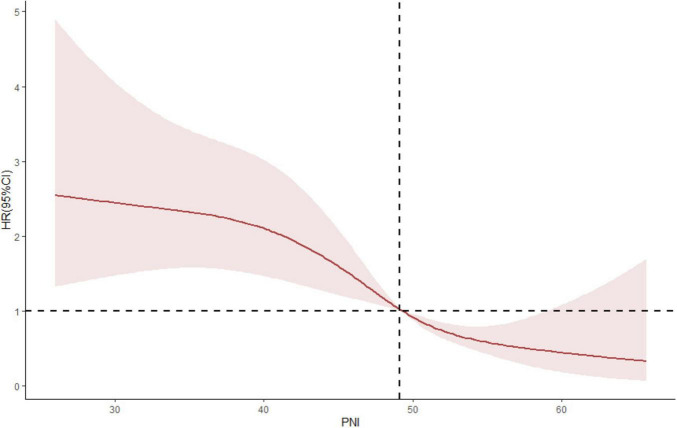
A non-linear association between PNI and renal outcome was found from restricted cubic spline (RCS) model.

PNI is a comprehensive index including serum albumin and blood lymphocyte count. We found that patients with low PNI had worse prognosis. Besides, non-linear association between PNI and renal outcome was found from restricted cubic spline (RCS) model, it also shown that in the process of increasing PNI, although the renal risk was still low, the upper 95% confidence interval indicated that the renal risk began to increase (Figure 6). Therefore, it is still necessary to further study which interval of PNI value can better predict the prognosis of patients in the future.

This study had two main limitations. First, the study’s retrospective nature might have distorted the results due to selection bias or missing data, and further multicenter retrospective studies are needed. Second, the mean follow-up time of 59 months was relatively short, especially for IgAN, which is a slowly progressing disease. We are continuing to follow up all patients to explore the long-term effect of this biomarker in the future.

In summary, our findings provide new evidence that PNI is an independent predictor of the risk of disease progression in patients with IgAN even after adjusting for traditional risk factors, such as hypertension, proteinuria, eGFR, anemia and hypoalbuminemia. PNI, which can be easily evaluated in daily clinical settings, as the constituents of PNI are common laboratory data, might be very important in the comprehensive management of IgAN patients.

## Conclusion

The PNI is a novel risk factor for predicting disease progression and prognosis in patients with IgAN.

## Data Availability

The raw data supporting the conclusions of this article will be made available by the authors, without undue reservation.

## Footnotes

1
http://www.rproject.org
